# 

**Published:** 1916-01-29

**Authors:** 


### CONTENTS.

| | PAGE |
|---|---|
| **The Termination of Honorary Staff Appointments** ... | 377 |
| **Possible Changes of the Future** ... | 378 |
| **Hospital and Institutional News** ... | 379 |
| The Florence Nightingale Memorial—Grave Illness of Miss Lückes—Truro's Naval Auxiliary Hospital—The Bradford War Hospital Scheme—Medical Attendance on Soldiers' Dependants—The Renovation of the Royal United Hospital, Bath—The Ambulance Department of the Order of St. John of Jerusalem—A Mental Hospital Scheme Held Up—The Health of Munition Workers—The Organisation of Ex-Patients' Subscriptions—Medicinal Herb Farming—The Selling of Diluted Milk—A New Institution at Droitwich—Serious Robbery at a London Hospital—The Payment of Hospital Clerks—A Strange Affair at Croydon Hospital—A Hospital Sunday School—The Lambeth-Southwark Dispute—Military Hospital Extensions in Leicester—A Cavell Cot at Stockport Infirmary—Alleged Nuisance at a Military Hospital—Honours for the Nursing Profession—Coventry and Warwickshire Hospital—The British Hospital at Petrograd—The College of Nursing—The "*Southern Cross*"—A Hospital for Neurasthenic Soldiers—Dispensary Finances in War-Time—An Interned Doctor's Tuberculosis Serum—This Week's Drug Market. | |
| **The Enteric Fevers in the War** ... | 385 |
| A Triumph of Scientific Medicine. | |
| **Research at the Seat of War** ... | 387 |
| The Combating of Wound Infections. | |
| **A Medical Causerie** ... | 389 |
| **Guy's Hospital and the War** ... | 390 |
| Making the Unfit into Serviceable Recruits. | |
| **National Insurance Act Developments** ... | 391 |
| Rules as to Behaviour during Sickness. | |
| **Abnormal Times and their Burdens** ... | 392 |
| **Hospital Architecture and Construction** ... | 393 |
| Coventry and Warwickshire Hospital. | |
| **Parliament and the Profession** ... | 394 |
| **Voluntary Work for Military Hospitals** ... | 395 |
| A Bournemouth Workshop. | |
| **Buildings Contemplated** ... | 395 |
| **Round the World** ... | 396 |
| Fellow-Workers and the Roll of Honour. | |
| **The Editor's Letter-Box** ... | 397 |
| Honours and the Nursing Profession—Robbing the Hospitals: The Homœopathic Hospital Case—The Western Hospital, Torquay—The Women's Maternity Unit for Russia. | |
| **Passing Events and Topics** ... | 398 |
| **Institutional Needs** ... | 398 |
| **The Institutional Worker** .. (Suppl.) | 1 |
| Co-operative Poultry Farming for Hospitals. | |

